# Comparing the effectiveness of dual-task and single-task training on walking function in stroke recovery: A systematic review and meta-analysis

**DOI:** 10.1097/MD.0000000000041776

**Published:** 2025-03-07

**Authors:** Weiyuan Tao, Jiawei Chen, Jiahui Peng, Wenwu Xiao

**Affiliations:** a Department of Rehabilitation Medicine, People’s Hospital of Yangjiang, Yangjiang City, Guangdong, China; b College of Sports Science, Shenyang Normal University, Shenyang City, Liaoning, China; c Faculty of Physical Education, National Research Tomsk State University, Tomsk City, Russia; d Department of Rehabilitation Medicine, The First Affiliated Hospital of Sun Yat-sen University, Guangzhou City, Guangdong, China.

**Keywords:** dual-task, rehabilitation, single-task, stroke

## Abstract

**Background:**

Stroke is a prevalent neurological disease with high morbidity and disability. Single-task walking training has limitations, and dual-task walking training has emerged. Yet, research on the relative effectiveness of dual- and single-task training for stroke patients’ walking function is inconclusive. This study aims to systematically compare the efficacy of dual-task with single-task training interventions on improving walking function among stroke survivors.

**Methods:**

A comprehensive search of electronic databases was conducted to identify randomized controlled trials investigating the application of dual-task training on walking function in stroke patients. Two reviewers independently screened the references, selected relevant studies, extracted data, and assessed the risk of bias. The primary outcome measures related to walking function included step speed, step length, stride length, step frequency, Berg balance scale (BBS), and timed up and go (TUG) test. The Cochrane risk of bias tool was used for methodological quality assessment of the included literature. Statistical analysis was performed using RevMan 5.4 software. Furthermore, the quality of evidence of the outcome measures was evaluated using the GRADEPro software.

**Results:**

A total of 17 studies were enrolled in this systematic review and meta-analysis. The results revealed that dual-task training exhibited significantly superior efficacy compared to single-task training in enhancing step speed, step length, stride length, step frequency, and BBS score (*P* < .05). However, no significant difference was observed in the TUG test (*P* = .100).

**Conclusion:**

Compared with traditional single-task training, dual-task training could be more effective in improving walking function among stroke patients, especially with regard to temporal and spatial parameters such as step length and speed, stride frequency and BBS score, but the effect on enhancing TUG test still remains unclear. These findings would help clinicians to formulate a more rational stroke rehabilitation strategy.

## 1. Introduction

Stroke is a prevalent neurological disease associated with high morbidity and disability. Impaired walking function is a common clinical symptom, exerting a substantial impact on patients’ quality of life, while also imposing a substantial economic burden on both families and society.^[1,2]^ Therefore, it is crucial to identify effective rehabilitation training methods to facilitate the walking function of stroke patients.

Single-task walking training, which has been a foundational component of stroke rehabilitation, primarily concentrates on the act of walking itself. Nevertheless, this approach may fail to sufficiently integrate the multifaceted cognitive and motor challenges inherent in everyday activities. Recognizing the limitations of single-task training, particularly its inadequate capacity to enhance attention and cognitive processing concurrent with walking, the development of dual-task walking training has emerged as a strategic advancement in addressing these complexities.^[3]^ This approach entails the concurrent execution of cognitive or motor tasks alongside walking training, thereby augmenting the intricacy and complexity of the intervention.^[4]^ By challenging patients to divide their attention between walking and another task, dual-task training aims to more closely mimic the multitasking nature of everyday activities, potentially leading to better functional outcomes and a reduced risk of falls.^[5]^

Although some studies have demonstrated that dual-task training interventions exhibit superior performance in enhancing walking function among stroke patients compared to single-task training,^[6,7]^ others have reported no significant disparities in gait and balance indices between these 2 methods,^[8]^ with certain studies even suggesting that dual-task training may be less effective than single-task.^[9]^

Given the inconsistent results of different studies, the aim of this study was to conduct a systematic review and meta-analysis to compare the effectiveness of dual-task and single-task training on walking function among stroke patients. Through this endeavor, the present study would provide robust evidence for selecting appropriate stroke rehabilitation methods, ultimately facilitating optimal promotion of walking function recovery in this population.

## 2. Materials and methods

This review was conducted according to the guidelines outlined in the Preferred Reporting Items for Systematic Reviews and Meta-analysis statement.^[10]^

### 2.1. Selection criteria

We included articles following these tips: type of study: randomized controlled trial (RCT). Subjects: patients with a clinical diagnosis of stroke^[11]^ and in the recovery period (≥3 months poststroke). Interventions: the experimental group used dual-task training while the control group only applied single-task training. The outcome indicators included: step speed (cm/s), step length (cm), stride length (cm), step frequency (step/min), and Berg balance scale (BBS) score.^[12]^ A higher value of the aforementioned relevant indicators represents better walking ability; timed up and go (TUG) test,^[13]^ with less time signifying better walking movement and balance function.

The exclusion criteria were: articles appeared only in abstract format, quasi-RCTs, no outcome indicators mentioned in the inclusion criteria, data could not be accurately extracted or missing from the original study, non-English studies, and inaccessibility of the full text.

### 2.2. Literature search strategies

We conducted a comprehensive literature search using PubMed, Web of Science, Cochrane Library, Scopus, and Embase databases. The search spanned from the inception of each database to October 2023 and employed a combination of subject headings (MeSH) terms and free terms. Additionally, we traced the references of the included studies to supplement the relevant literature and expand the number of sources. The specific search strategy used for PubMed was as follows: (“Stroke”[MeSH Terms] OR “cerebral vascular” OR “poststroke”) AND (“dual task” OR “dual-task” OR “cognitive motor” OR “cognitive-motor” OR “motor cognitive” OR “motor-cognitive” OR “additional task”) AND (“Gait”[MeSH Terms] OR “walk” OR “walking”).

Search strategy for Web of Science: ((TS = (“Stroke” OR “cerebral vascular” OR “poststroke”)) AND TS = (“dual task” OR “dual-task” OR “cognitive motor” OR “cognitive-motor” OR “motor cognitive” OR “motor-cognitive” OR “additional task”)) AND TS= (“Gait” OR “walk” OR “walking”).

Search strategy for Cochrane Library: #1: Stroke (84595). #2: (cerebral vascular):ti,ab,kw OR (poststroke):ti,ab,kw (9216). #3: #1 OR #2 (86108). #4: (dual task):ti,ab,kw OR (dual-task):ti,ab,kw OR (cognitive motor):ti,ab,kw OR (motor cognitive):ti,ab,kw (Word variations have been searched) (10555). #5: (walk):ti,ab,kw OR (walking):ti,ab,kw OR (gait):ti,ab,kw (Word variations have been searched) (48162). #6: #3 AND #4 AND #5 (206).

Search strategy for Scopus: (TITLE-ABS-KEY (“Gait”) OR TITLE-ABS-KEY (“walk”) OR TITLE-ABS-KEY (“walking”)) AND (TITLE-ABS-KEY (“Gait”) OR TITLE-ABS-KEY (“walk”) OR TITLE-ABS-KEY (“walking”)) AND (TITLE-ABS-KEY (“Stroke”) OR TITLE-ABS-KEY (“cerebral vascular”) OR TITLE-ABS-KEY (“poststroke”)).

Search strategy for Embase: (stroke OR “cerebral vascular” OR poststroke) AND (“dual task” OR “cognitive-motor” OR “cognitive motor” OR “motor-cognitive” OR “motor cognitive” OR “additional task”) AND (gait OR walk OR walking).

Literature screening: The databases were searched based on the developed search strategy. Then titles and abstracts of the identified literature were initially screened to exclude irrelevant studies according to the inclusion criteria. Subsequently, the full-text review was conducted for further screening, and any literature that did not meet the criteria were excluded. The screening process was carried out independently by 2 researchers, and any discrepancies were resolved through discussion or consultation with a third reviewer.

Data extraction: Basic information of the included studies: such as authors’ names, year of publication; population characteristics, including sample size, mean age, and gender proportion; intervention method, training intensity, and duration; and outcome measures of step speed, step length, stride length, step frequency, BBS scores, and TUG test.

### 2.3. Risk of bias assessment of included studies

The risk of bias of the included studies was evaluated using the risk of bias assessment tool for RCTs, as recommended by the Cochrane Handbook (version 5.1.0).^[14]^ The specific details of the evaluation included: whether the randomization process was conducted appropriately; whether the allocation sequence was adequately concealed; whether the participants, intervention providers, and outcome assessors were effectively blinded; whether the outcome data could be extracted completely; whether the results were reported selectively; and identification of any other potential sources of bias. Two independent researchers conducted the risk of bias assessment with cross-checking to ensure accuracy. Any discrepancies were resolved through consultation or by involving a third reviewer.

### 2.4. Statistical analysis

Meta-analysis was performed using the R software and the RevMan 5.4 software. The outcome indicators included in this paper were all continuous variables, and each indicator was tested by the same unit, so the mean difference (MD) was selected as the effect indicator. The *P* value was used for the heterogeneity test. When *P* ≥ .1, there was no heterogeneity or low heterogeneity; a fixed-effect model was used for analysis. When *P* < .1, with high heterogeneity, a random effect model was used for analysis. In addition, subgroup analysis and risk of bias were also discussed for the main indicators.

## 3. Results

### 3.1. Literature screening process and results

**Figure 1.**
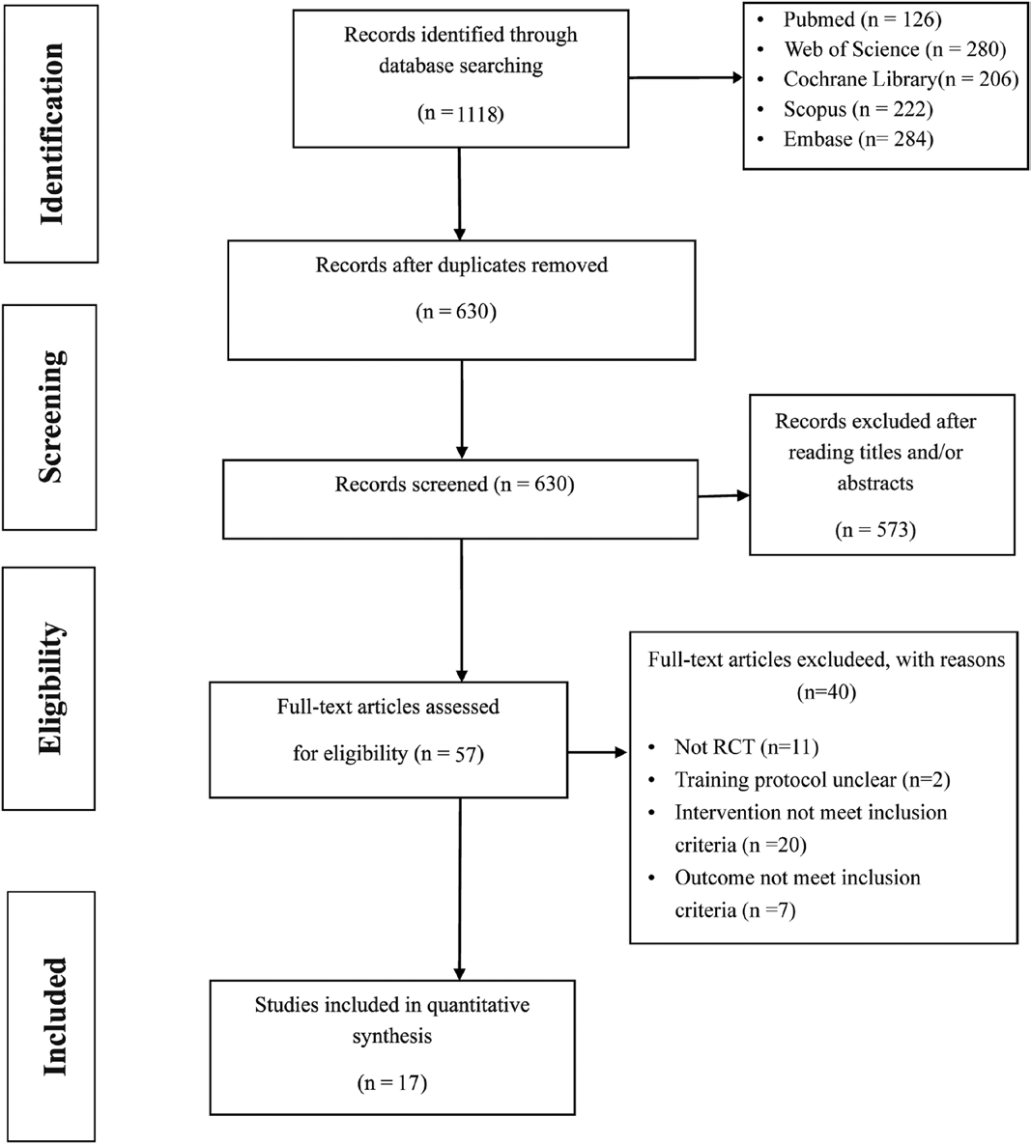
Flow chart of study selection. RCT = randomized controlled trial.

A total of 1118 papers were initially screened, resulting in 630 papers after removing duplicates. Subsequently, upon reviewing the titles and abstracts, 57 papers were selected for further evaluation. Finally, following a comprehensive full-text rescreening process according to the Preferred Reporting Items for Systematic Reviews and Meta-analysis guidelines, which was summarized in Figure 1, a total of 17 RCTs^[15–31]^ involving 636 patients were included.

### 3.2. Basic characteristics and risk of bias assessment results

**Figure 2.**
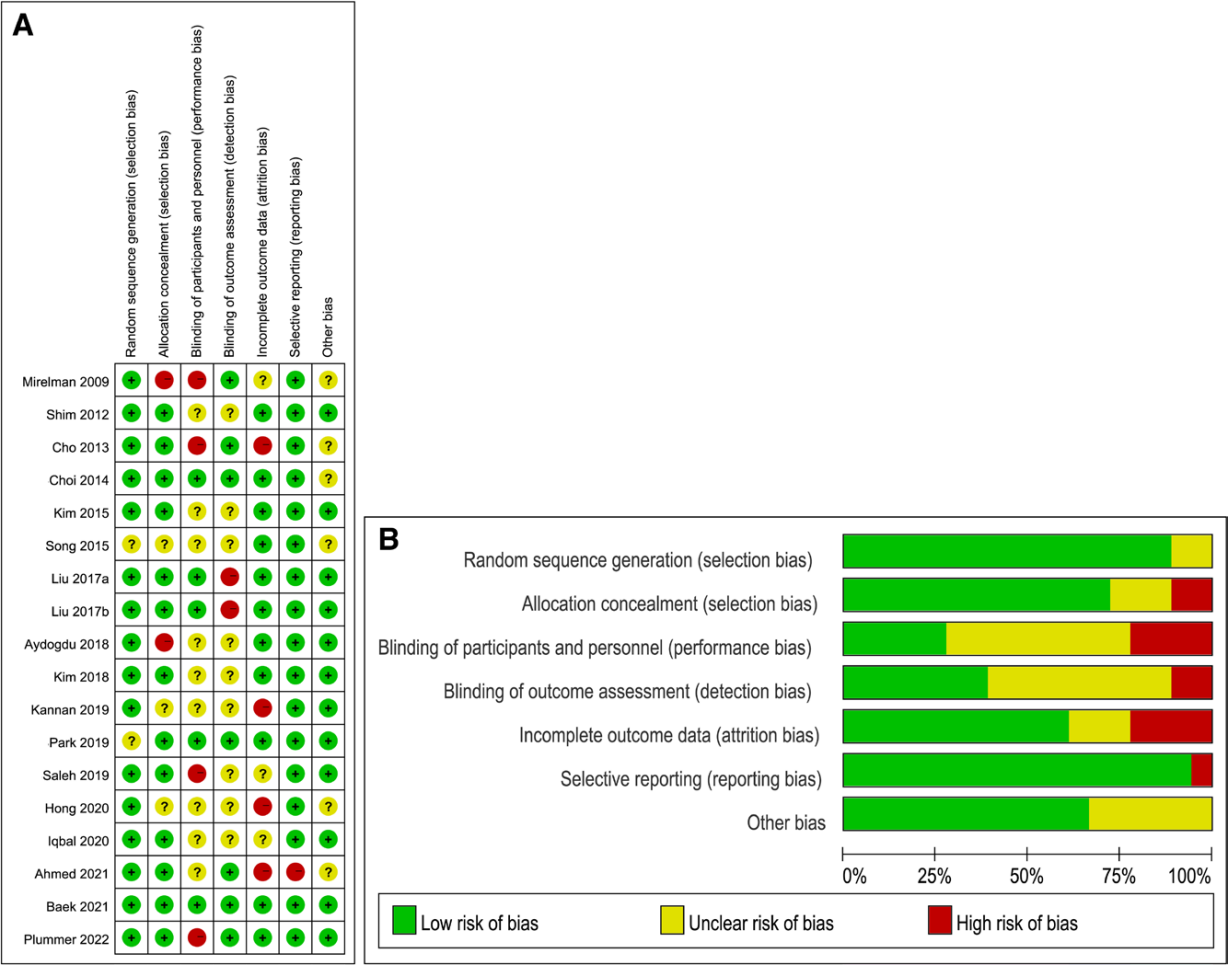
(A) Risk of bias summary. (B) Risk of bias graph.

The basic characteristics of the included studies were presented in Table 1. The risk and bias assessment of the literature was evaluated by the Cochrane Risk of Bias Assessment Tool,^[14]^ and the results were illustrated in Figure 2A and B.

**Table 1:** Characteristics of included studies.

Study	Year	Time after stroke	Sample size		Age		Gender		Treatment		Training duration	Training intensity	Outcome
			Experiment	Control	Experiment	Control	Female	Male	Experiment	Control			
Mirelman et al, 2009^[15]^	2009	Chronic	9	9	61.80	61.00	3	15	Cognitive dual task gait training	Single-task training	4 wk	60 min per session 3 sessions per wk	Walk speed
Shim et al, 2012^[16]^	2012	Chronic	17	16	65.59 ± 5.81	61.56 ± 6.17	13	20	Motor dual-task training	Single-task training	6 wk	30 min, 3 d/wk	Walk speed; step length; step frequency
Cho et al, 2013^[17]^	2013	Chronic	7	7	64.57	65.14	7	7	Cognitive dual-task gait training	Single-task training	6 wk	30 min per session 3 sessions per wk	Walk speed; step length; step frequency; stride length; BBS; TUG test
Choi et al, 2014^[18]^	2014	Chronic	19	18	49.11 ± 11.93	49.33 ± 8.27	31	6	Cognitive-motor dual-task with a random auditory cue while walking on a treadmill	Single-task gait training with treadmill	4 wk	15 min per session 5 sessions per wk	BBS; TUG test
Kim et al, 2015^[19]^	2015	Chronic	20	20	51.00	48.10	14	26	Cognitive dual-task gait training	Single-task gait training with treadmill	4 wk	30 min per session 3 sessions per wk	Walk speed; step length; step frequency; stride length
Song et al, 2015^[20]^	2015	Chronic	20	20	55.37 ± 20.6	57.10 ± 7.80	19	21	Motor dual-task training	Single-task training	8 wk	30 min/d 5 times per wk	BBS
Liu et al,2017a^[21]^	2017	Chronic	9	10	51.00 ± 7.10	50.80 ± 13.50	3	16	Cognitive dual-task gait training	Single-task training; conventional physical therapy	4 wk	30 min per session 3 sessions per wk	Walk speed; step length; step frequency; stride length
Liu et al, 2017b^[21]^	2017	Chronic	9	10	48.80 ± 11.70	50.80 ± 13.50	4	24	Motor dual-task gait training	Single-task training	4 wk	30 min per session 3 sessions per wk	Gait speed; step frequency
Aydogdu et al, 2018^[22]^	2018	Chronic	25	28	71.21 ± 4.92	69.28 ± 5.03	14	39	Cognitive-motor dual-task gait training	Single-task gait training	4 wk	30 min per session 5 sessions per wk	BBS
Kim et al, 2018^[23]^	2018	Chronic	13	13	52.62	56.20	11	15	Cognitive dual-task gait training	Single-task gait training with treadmill	4 wk	30 min per session 5 sessions per wk	Walk speed; step length; step frequency
Kannan et al, 2019^[24]^	2019	Chronic	13	11	57.50 ± 8.04	61.00 ± 4.60	11	13	Cognitive-motor exergame training	Single-task training	6 wk	5 session for 1–2 wk, 3 session for 3–4 wk, 2 session for 5–6 wk, and 90 min each	TUG test; BBS
Park et al, 2019^[25]^	2019	Chronic	15	15	56.30 ± 7.14	59.75 ± 7.75	16	24	Dual-task using different cognitive tests	Single-task training	6 wk	30 min per session 3 sessions per wk	Walk speed; step frequency
Saleh et al, 2019^[26]^	2019	Chronic	25	25	49.53 ± 1.8	50 ± 1.96	26	24	Motor dual-task training	Motor single-task training	6 wk	45 min per session 3 sessions per wk	Step length
Hong et al, 2020^[27]^	2020	Chronic	8	9	49.11 ± 11.93	49.33 ± 8.27	7	10	Cognitive task training	Single-task training	4 wk	3 times per wk, 30 min per time	Walk speed
Iqbal et al, 2020^[28]^	2020	Chronic	32	32	58.28 ± 7.13	58.87 ± 6.13	30	34	Motor dual-task training	Motor single-task training	4 wk	40 min per session 4 sessions per wk	Step length; step frequency
Baek et al, 2021^[29]^	2021	Chronic	16	15	56.94 ± 8.79	56.13 ± 10.25	11	20	Dual-task gait training with treadmill	Single-task gait training with treadmill	6 wk	30 min per session 2 sessions per wk	Walk speed; step frequency
Ahmed et al, 2021^[30]^	2021	Chronic	42	42	61.21 ± 7.78	62.21 ± 8.20	47	37	Intensive multiplanar trunk training coupled with dual-task exercises	Single-task training	12 wk	45 min per session 5 sessions per wk	TUG test; BBS
Plummer et al, 2022^[31]^	2021	Chronic	18	19	54.40 ± 16.40	59.60 ± 14.50	17	19	Dual-task gait training	Single-task gait training	4 wk	30 min per session 3 times per wk	Walk speed; TUG test

BBS = Berg balance scale, TUG test = timed up and go test.

### 3.3. Gait indicators

**Figure 3.**
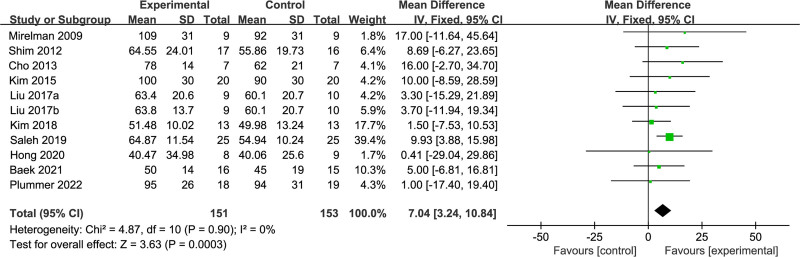
Comparison of step speed between dual-task group and single-task group. CI = confidence interval, SD = standard deviation.

In a cohort of 304 stroke patients, step speed was evaluated in 10 RCT studies.^[15–17,19,21,23,26,27,29,31]^ The calculated *I*^2^ value was 0%, indicating a high degree of statistical homogeneity. Given the high uniformity, a fixed-effects model was appropriate for meta-analysis. The findings revealed a notably higher step speed in the dual-task group than the single-task group (MD = 7.04; 95% confidence interval [CI]: 3.24–10.84; *P* < .001). More details can be found in Figure 3.

**Figure 4.**
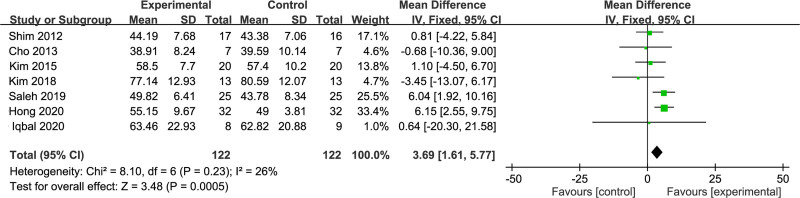
Comparison of step length between dual-task group and single-task group. CI = confidence interval, SD = standard deviation.

Additionally, step length as an outcome parameter was probed in 7 RCT studies^[16,17,19,23,26–28]^ involving 244 stroke patients. The *I*^2^ value amounted to 26%, representing good homogeneity. A fixed-effects model was used in conducting the meta-analysis. The results displayed a longer step length in the dual-task group than the single-task group (MD = 3.69; 95% CI: 1.61–5.77; *P* < .001). Figure 4 provides a visualization of these outcomes.

**Figure 5.**
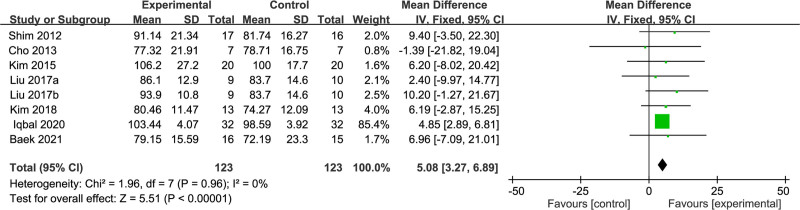
Comparison of step frequency between dual-task group and single-task group. CI = confidence interval, SD = standard deviation.

Moreover, step frequency was investigated as an outcome measure in 7 RCT studies^[16,17,19,21,23,28,29]^ with an aggregate of 246 stroke patients. Given an *I*^2^ value of 26%, high homogeneity among the study results was established with the fixed-effects model for meta-analysis. The interpretation exhibited a higher step frequency in the dual-task group than the single-task group (MD = 5.08; 95% CI: 3.27–6.89; *P* < .001). Further details are presented in Figure 5.

**Figure 6.**
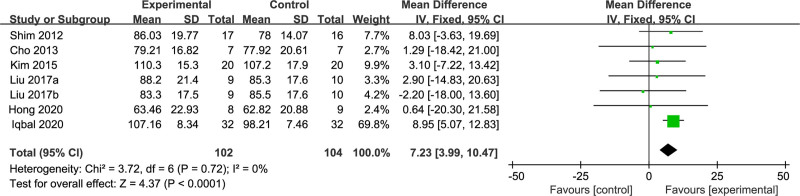
Comparison of stride length between dual-task group and single-task group. CI = confidence interval, SD = standard deviation.

Finally, stride length was analyzed as an outcome parameter in 6 RCT studies^[16,17,19,21,27,28]^ with a total of 206 stroke patients. The *I*^2^ value was 0%, which suggested substantial homogeneity among the results. We used a fixed-effects model for meta-analysis and found that the stride length was greater in the dual-task group compared to the single-task group (MD = 7.23; 95% CI: 3.99–10.47; *P* < .001), as illustrated in Figure 6.

### 3.4. Balance indicators

**Figure 7.**
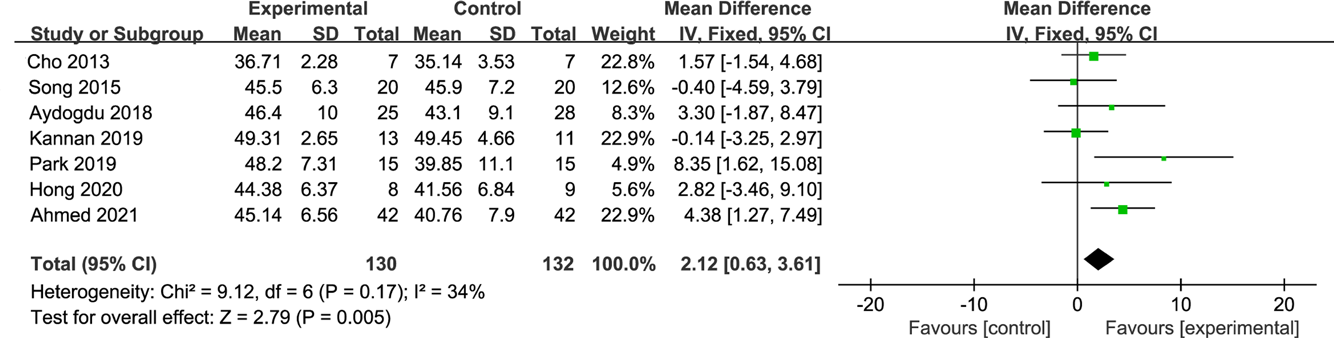
Comparison of Berg balance scale scores between dual-task group and single-task group. CI = confidence interval, SD = standard deviation.

A total of 7 RCT studies^[17,20,22,24,25,27,30]^ utilized BBS score as an outcome measure, involving 262 stroke patients. The *I*^2^ value was calculated to be 34%, demonstrating good homogeneity. A fixed-effects model was chosen for meta-analysis, and the results indicated that the BBS score of the dual-task group was significantly better than that of the single-task group (MD = 2.12; 95% CI: 0.63–3.61; *P* = .005), as shown in Figure 7.

**Figure 8.**
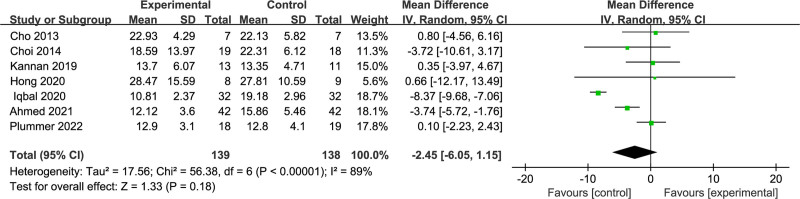
Comparison of timed up and go test between dual-task group and single-task group. CI = confidence interval, SD = standard deviation.

Furthermore, 7 RCT studies^[17,18,24,27,28,30,31]^ used the TUG test as an outcome indicator, encompassing 277 stroke patients. Given an *I*^2^ value of 89%, high heterogeneity among the study results was established with the random-effects model for meta-analysis. The findings revealed that there was no difference in the TUG test between the dual-task group and the single-task group (MD = −2.57; 95% CI: −5.64 to 0.50; *P* = .100), as described in Figure 8.

To further investigate potential sources of heterogeneity, subgroup analyses were conducted on potential moderating variables (Table 2). Subgroup analyses based on the type of intervention revealed that neither the motor-cognitive dual-task intervention nor the motor dual-task intervention had a significant impact on improving the TUGT performance of subacute stroke patients. However, the motor dual-task group exhibited considerable heterogeneity. Similarly, subgroup analyses based on intervention frequency demonstrated that both ≤ 3 times per week and > 3 times per week interventions did not yield significant improvements in the TUGT performance of subacute stroke patients. Notably, the > 3 times per week group displayed substantial heterogeneity. Moreover, subgroup analyses based on the number of interventions indicated that both < 20 times and ≥ 20 times interventions did not result in significant improvements in the TUGT performance of subacute stroke patients. Interestingly, the < 20 times group showed marked heterogeneity. Finally, subgroup analyses based on the duration of each intervention session revealed that both ≤ 30 and > 30 minutes interventions did not lead to significant improvements in the TUGT performance of subacute stroke patients. Notably, the > 30 minutes group exhibited notable heterogeneity.

**Table 2:** Subgroup analysis of the TUG test between dual-task group and single-task group.

Variables	Type of subgroup	n/RCTs	Heterogeneity summary			Subgroup meta-analysis summary		
			Q	*P*	*I*^2^/%	Cohen d	95% CI	*P*
Method	Cognitive-motor	4	1.01	.80	0.00	−0.09	−0.50 to 0.32	.679
	Motor dual-task	2	42.81	.00	96.43	−1.29	−3.12 to 0.54	.166
Frequency	≤3 times/wk	4	0.04	.10	0.00	0.06	−0.35 to 0.47	.772
	>3 times/wk	3	36.25	.00	95.68	−1.41	−3.08 to 0.26	.997
Times	<20 times	4	51.27	.00	93.34	−0.74	−2.33 to 0.85	.362
	≥20 times	3	3.93	.14	49.25	−0.45	−0.95 to 0.05	.079
Per time	≤30 min	4	1.00	.80	0.00	−0.08	−0.46 to 0.30	.681
	>30 min	2	39.07	.00	95.88	−1.29	−3.13 to 0.56	.171

CI = confidence interval, RCT = randomized controlled trial, TUG test = timed up and go test.

### 3.5. Publication bias analysis

**Figure 9.**
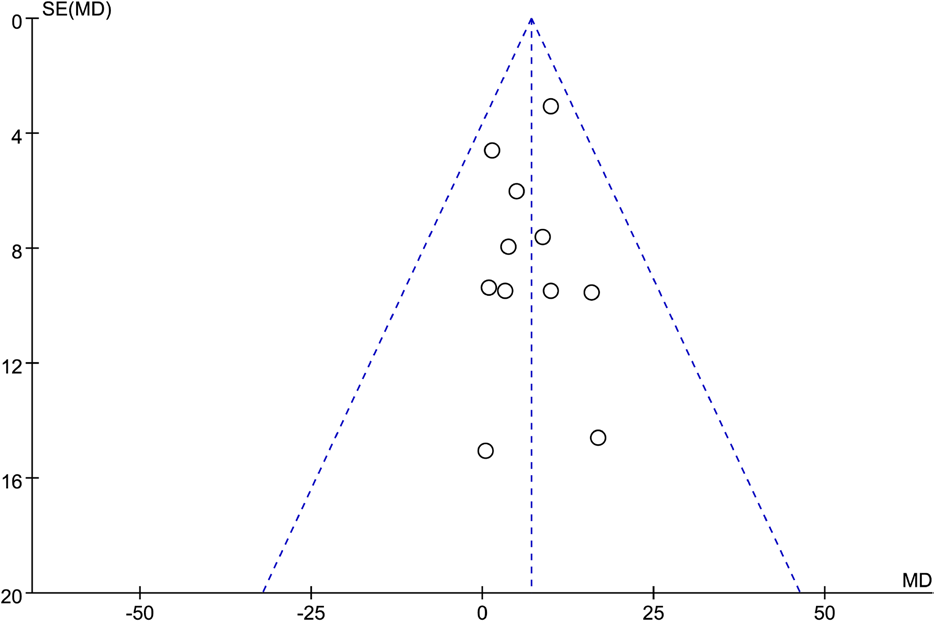
Funnel plot about step speed. MD = mean difference.

From the funnel plot (Fig. 9), it can be seen that the graph is basically symmetrical. The result suggests no publication bias in the study.

As is known to all, funnel plots were not recommended to use for publication bias analysis when the studies included <10.^[32]^ We conducted a subjective publication bias analysis, resulting in the following observations: the inclusion of RCTs in this study with a smaller sample size might increase the risk of publication bias.^[33]^ The inclusion of only English-language research in this study could induce certain publication bias either.

### 3.6. Evidence quality assessment

**Figure 10.**
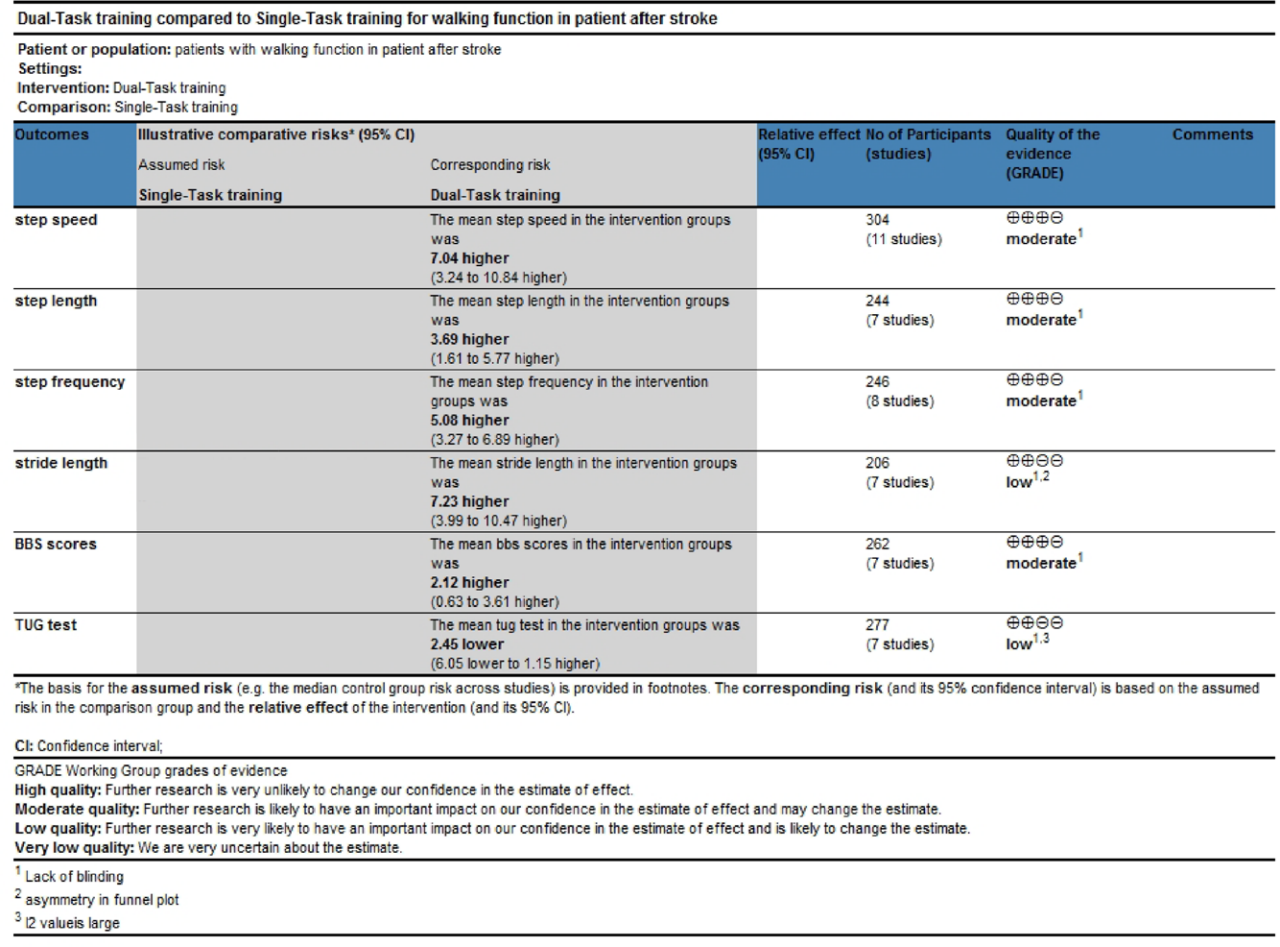
Evidence quality assessment.

The GRADEPro software indicated that the evidence quality for step speed, step length, step frequency, and BBS scores was rated as moderate. However, for stride length and the TUG test, the evidence quality was low, as shown in Figure 10.

### 3.7. Adverse events

Inclusion of 17 pieces of literature did not report any adverse events occurring in studies.

## 4. Discussion

Dual-task training refers to the concurrent performance of a primary task along with a secondary task, which is typically categorized into cognitive-motor and motor-motor dual-task models, and is increasingly utilized in stroke rehabilitation.^[34–36]^ However, there are still conflicting findings regarding the comparative efficacy of dual-task training versus traditional single-task training in improving walking function.

Traditional rehabilitation training methods primarily focus on the recovery of independent walking ability under single-task conditions, aiming to enhance the patient’s local-motor ability. However, integrating dual-task situations into the rehabilitation process has the potential to be more effective in improving the motor walking function of stroke patients.^[37]^ The results of our meta-analysis demonstrated significant differences in step speed, step length, stride length, and step frequency in the dual-task group, compared with the control group. Several potential reasons for these findings were identified. First, dual-task training has the potential to optimize cognitive resource allocation, facilitating a balanced distribution of attention between primary and secondary tasks. This optimization could lead to improved movement coordination, enhanced gait-related temporal-spatial parameters, and ultimately result in advanced walking function.^[38]^ Second, the movement pattern in a dual-task context tends to become more automated, reducing the influence of limited attentional resources on postural control.^[39]^ Thirdly, dual-task training could further enhance sensorimotor perceptual integration and activate brain regions related to central executive function, such as the dorsolateral prefrontal cortex, left inferior frontal gyrus, anterior cingulate cortex, precuneus, and cerebellum.^[40]^ These mechanisms would promote endogenous neural repair, increase the number of neuronal synapses in the cerebral cortex, and facilitate the lateral sprout regeneration of axons and dendrites, thereby improving the nervous system’s ability to control the body and enhancing patient’s walking function.^[41]^ Additionally, adding cognitive training alongside motor tasks could effectively strengthen the brain’s functional network connections, making the coordinated interoperability of motor-cognitive-related neural resources more efficacious and promoting the improvement of walking motor ability.^[42]^

In this study, the BBS scores exhibited better performance in the dual-task training group compared with the single-task group. The reason for this refinement might be that the dual-task training intervention could enhance gait parameters in stroke patients, such as step speed, stride length, and step frequency. As a result of these improvements, the percentage of the double support phase was reduced, and the single support phase of the hemiplegia side leg was relatively prolonged.^[43]^ This led to higher balance demands during the dynamic walking process, ultimately resulting in a superior performance of BBS.

Interestingly, this meta-analysis showed no differences in the TUG test between the dual-task and the single-task groups. Several potential reasons were identified. First, compared with straight-line walking assessment, the TUG test demands more function to assess balance, gait speed, and lower limb strength simultaneously.^[44]^ Although it is suitable for overall walking functional assessments in daily life, its validity in stroke patients may not be credible.^[44]^ Second, dual-task training attempts to improve the rehabilitation effect through the redistribution of attentional resources. However, there is a lack of reported analysis regarding the distribution of motor and cognitive tasks within dual-task training, impeding quantitative assessment of neural resource allocation. Moreover, the cognitive task during the dual-task training process may be relatively easy and occupy fewer neural resources,^[45]^ which may not fully capture the distinctions between the 2 training methods. In subgroup analysis, although the *P* values in the motor dual-task, > 3 times per week, < 20 times, and > 30 minutes among the subanalysis of the TUG test were >.05, the heterogeneity was high. Therefore, these results could not determine which training type could better improve TUGT performance under these conditions. Future researchers are required to do more high-quality randomized controlled experiments in this motor element condition to verify the difference between these 2 training types in improving TUGT performance.

It was important to note that this meta-analysis did not contain non-English studies, and most of the included studies did not explicitly describe the blinding method, which might bring a certain risk of publication bias. Additionally, the content of motor single-task training in the included studies broadly focuses on gait training, balance training, and strength training. The specific effect could depend on the level of competence or implementation of each unit or healthcare department, which might have a certain impact on the results. Furthermore, this study aimed to compare the differences between dual-task and single-task training, but not the differences of dual-task types, which deserves further comparative analysis.

## 5. Conclusion

In summary, the current evidence suggests that dual-task training is more effective than traditional single-task training in walking function rehabilitation of stroke, particularly in step speed, step length, step frequency, stride length, and BBS score. However, the superiority of dual-task training in improving TUG test requires further exploration. These findings provide valuable references for future clinical practice and contribute to the development of a more rational stroke rehabilitation strategy. However, due to the limitation of the number and quality of included studies, the above findings still require validation from additional high-quality research.

## Acknowledgments

The authors would like to express their gratitude to the participants and staff involved in data collection and management in the database.

## Author contributions

**Data curation:** Weiyuan Tao, Jiawei Chen, Jiahui Peng, Wenwu Xiao.

**Writing – original draft:** Weiyuan Tao.

**Writing – review & editing:** Jiawei Chen.

**Software:** Jiahui Peng.

**Visualization:** Jiahui Peng.

**Methodology:** Wenwu Xiao.
